# Matrix Mechanosensation in the Erythroid and Megakaryocytic Lineages

**DOI:** 10.3390/cells9040894

**Published:** 2020-04-06

**Authors:** Christina M. Ward, Katya Ravid

**Affiliations:** 1 Department of Medicine and Whitaker Cardiovascular Institute, Boston University School of Medicine, Boston, MA 02118, USA; cmward@bu.edu; 2 Departments of Biochemistry and Biology, Boston University School of Medicine, Boston, MA 02118, USA

**Keywords:** extracellular matrix, megakaryocyte, erythroid lineage, integrins, mechanosensitive ion channels

## Abstract

The biomechanical properties of the bone marrow microenvironment emerge from a combination of interactions between various extracellular matrix (ECM) structural proteins and soluble factors. Matrix stiffness directs stem cell fate, and both bone marrow stromal and hematopoietic cells respond to biophysical cues. Within the bone marrow, the megakaryoblasts and erythroblasts are thought to originate from a common progenitor, giving rise to fully mature magakaryocytes (the platelet precursors) and erythrocytes. Erythroid and megakaryocytic progenitors sense and respond to the ECM through cell surface adhesion receptors such as integrins and mechanosensitive ion channels. While hematopoietic stem progenitor cells remain quiescent on stiffer ECM substrates, the maturation of the erythroid and megakaryocytic lineages occurs on softer ECM substrates. This review surveys the major matrix structural proteins that contribute to the overall biomechanical tone of the bone marrow, as well as key integrins and mechanosensitive ion channels identified as ECM sensors in context of megakaryocytosis or erythropoiesis.

## 1. Introduction

### 1.1. Overview

The bone marrow (BM) is the predominate location for adult hematopoiesis—the process in which a population of multipotent hematopoietic stem cells (HSCs) produce lineage-restricted progenitors that give rise to all the types of blood cells. The BM microenvironment is complex and supports the proliferation and differentiation of hematopoietic stem progenitor cells (HSPCs) through cues received from the extracellular matrix (ECM). The ECM is a non-cellular support network composed of structural proteins and various soluble factors [1,2]. As the physical support structure for the surrounding HSPCs, the ECM has a role in biological functions, such as adhesion, migration, apoptosis, proliferation, and differentiation [3]. The composition of the ECM is derived from a mixture of collagens; laminins; fibronectin and fibrinogen; and soluble proteins, such as cytokines, chemokines, and secreted enzymes. These various structural matrix proteins set the elasticity and rigidity of the bone marrow that creates the biophysical state surrounding the cells [1,2,3]. The ability of a cell to sense forces such as compression, tension, fluid shear stress, and hydrostatic pressure within the three-dimensional environment is a conversed ability that all single celled organisms and complex multicellular eukaryotes possess [3]. Characterizing the functional link from the biomechanical cues a cell receives to a biochemical response is the process called mechanotransduction [3]. This review focuses on the biomechanical properties of the BM compartment and how these properties influence the cell fate of the erythroid and megakaryocyte (MK) lineages through the process of mechanotransduction.

### 1.2. Bone Marrow ECM Composition

BM stromal and hematopoietic progenitor cells have been demonstrated to be mechanically responsive to engineered substrates and surrounding viscosity [4,5,6,7,8]. The rigidity of the BM is not uniform throughout, as there exists a large amount of heterogeneity in the Young’s modulus ranging from 0.25 to 24.7 kPa [9]. Several key matrix structural proteins contribute to the overall biomechanical tone of the bone marrow. The ECM is mainly made up of collagens, which include both fibrillary collagen (collagen I, II, III, V, and XI) and non-fibrillary collagen [10]. Immunohistochemical analysis of bone marrow revealed that collagen I and IV along with fibronectin are localized throughout the endosteum [11]. Multicolor quantitative confocal imaging cytometry, a technique that has allowed for a three-dimensional map of the ECM components, further confirmed that collagen I and fibronectin are pervasively localized throughout the entire bone marrow [12]. Furthermore, collagen IV, laminin, and fibronectin are largely present in the BM parenchyma [12]. This agrees with other sources that have also mapped type IV collagen, laminin, and fibronectin to the sinusoids [13]. The presence of collagen throughout the bone marrow contributes to the overall stiffness of the ECM [1,10]. The increase in stiffness is non-linear in response to increased deposition of collagen III mixed with collagen I [14,15]. Fibronectin, a glycoprotein, modulates ECM stiffness by organizing collagen into fibrils [16]. A collagen matrix failed to develop in fibronectin-deficient mouse fibroblasts that were cultured, but the addition of exogenous fibronectin reconstituted the collagen matrix [16].

The BM ECM is continuously subjected to remodeling by proteins such as matrix metalloproteinases (MMP), tissue inhibitors of MMPs (TIMPs), and plasmins. MMPs are a family of zinc-dependent endopeptidases which are responsible for the breakdown of the ECM, while TIMPs counterbalance the function of MMPs [17]. In patients with essential thrombocythemia and polycythemia vera plasma levels of MMP-3 were decreased and plasma TIMP-1 was elevated [18]. These data suggest that altered BM ECM composition contributes to these disease states. Endosteal expression of TMIP-3 promotes HSC regeneration and actively drives HSCs out of the quiescent state [19,20]. This is consistent with other studies that demonstrate that ECM substrate stiffness that resembles the endosteal space promotes HSC proliferation [8]. Plasmin is a protein that breaks down ECM fibrin, fibronectin, and laminin, and activates MMPs [21]. Urokinase plasminogen activator (uPA), which is broadly expressed by BM cells, actives plasmin [21,22,23]. Active plasmin is regulated by plasminogen activator inhibitor 1 (PAI-1) and tissue plasminogen activator (tPA) [21]. In the BM the expressions of PAI-1 and tPA offset one another functionally and are important for hematopoietic regeneration, particularly after BM transplantation [24,25]. Inhibition of PAI-1 improved hematopoietic recovery in mice after myeloablation. Taken together, the structural protein makeup of the BM ECM and the factors regulating these proteins are influential on the overall rigidity of the BM.

### 1.3. Effect of BM Stiffness on Cell Differentiation

ECM stiffness is important to the determination of HSC fate and differentiation [4,8,26]. HSCs cultured on stiffer matrices promoted the development of myeloid progenitors, while softer laminin surfaces increased the number of erythroid precursors [8]. Rigidity influences BM stromal cell differentiation [27]. Osteogenic differentiation of BM-derived mesenchymal stromal/stem cells (MSCs) is enhanced on stiffer substrate surfaces [27]. ECM stiffness modulates MK development and platelet production. When BM progenitors were cultured in 3D methylcellulose hydrogels designed to recapitulate the BM microenvironment (30–60 Pa), MKs had more demarcation membranes, higher ploidy, and produced more platelets compared to liquid cultures or stiffer 3D cultures (300–600 Pa) [28]. This was further corroborated by another study, wherein MKs were cultured on soft collagen-coated gels (300 Pa) or on stiffer gels (34 kPa) [29]. These cultured MKs had higher ploidy on the softer gels regardless of collagen concentration [29]. In fact, matrix stiffness seems to mediate MK differentiation and platelet production independent of the type of collagen. In an ex vivo model, cultured MKs produced more platelets on softer matrices regardless of whether the matrix was composed of collagen I or collagen IV [30,31]. β1 integrin was more highly activated on the softer matrix, and platelet formation on the softer silk was mediated through the PI3K/Akt pathway [30].

## 2. Integrins as ECM Sensors in the Context of Erythropoiesis

Integrins are heterodimeric type I transmembrane proteins composed of α and β subunits and serve as one of the major cell adhesion receptors in the ECM. Integrins act as a transmembrane link between the ECM and the cytoskeleton. The 24 mammalian integrin receptors are made up of a combination of 18 α and 8 β subunits [32]. Each subunit has a single transmembrane domain, a short cytoplasmic tail, and an extracellular domain that binds ligands such as collagen or fibronectin. [32]. Integrins on the surface of blood cells and platelets are inactive in a bent confirmation, and activation is triggered by variety of signaling molecules such as growth factors [32]. Integrin-ECM binding activates a multitude of intracellular pathways involved in fundamental biological processes, such as cell survival and proliferation, cytoskeletal organization, and cell mobility [32]. Knockout of integrin β1 in mice is lethal [33]. However, α2 integrin knockout mice are viable and develop normally, but platelet aggregation in these mice is delayed [34]. Integrin α4 is required to maintain normal hematopoiesis [35], and lack of α4 integrin disrupts T-cell and B-cell production, whereas monocyte and natural killer cell production remains unaffected by α2 knockout [36]. Mutations in integrin β3 lead to the disorder Glanzmann’s thrombasthenia, wherein platelets cannot bind fibrinogen and fail to aggregate [37]. β3 mutations contribute to cardiovascular disease and the development of atherosclerosis [37]. Integrins are essential for mechanosensing of the ECM and related signaling.

Erythropoiesis, the process in which progenitors undergo terminal differentiation into mature circulating red blood cells (RBCs), occurs in a specialize bone marrow structure called the erythroblastic island, which is characterized by a central macrophage surrounded by committed erythroid progenitors. Integrins are crucial to this process as they not only facilitate a connection to the surrounding ECM but mediate cell to cell contact between the central macrophage and erythroid progenitors. The predominate integrins expressed on erythroid progenitors are VLA-4 (α4β1) and VLA-5 (α5β1); both are fibronectin receptors [38]. While erythroid maturation is mediated considerably through growth factor signaling, principally through erythropoietin (EPO), adhesion to extracellular fibronectin and laminin promotes erythroid development [8,38]. The developmental expression of α4 can be used as a helpful tool to identify erythroid cells at different maturation states, as α4 expression decreases as the erythroid progenitors mature into terminally differentiated reticulocytes cells [39,40,41]. The decreased ability of erythroid cells to bind fibronectin correlates with the expression pattern of α4 during development; with early human erythroid progenitors (CFU-E) adhering to fibronectin, the intermediate progenitor (proerythroblast) has a diminished capability to bind to fibronectin, and terminal differentiated reticulocytes are unable to bind fibronectin [39]. This is necessary so that mature RBCs can exit the bone marrow and enter circulation. The effects of erythroid adhesion to fibronectin through integrins are temporally distinct as early ex vivo fetal liver erythroid progenitors require stimulation with erythropoietin (EPO), but fibronectin was found to be crucial for differentiation of late progenitors [38]. Erythroid progenitors’ engagement with fibronectin can be perturbed with the use of monoclonal antibodies against VLA-5 [39]. Inhibiting VLA-4 engagement with fibronectin blocked erythroid development; however, blocking VLA-5 in the same fashion did not have the same affect [42]. The roles of the two predominate fibronectin integrins on erythroid progenitors are debated, as it has not always been clear from in vivo models what distinct functions these fibronectin-binding integrins serve [43,44,45]. This raises the possibility that the stiffness of fibronectin used in the different studies could influence the effect of these integrins on erythroid differentiation. Several integrin knockout mice were generated and administered phenyl hydrazine which induces severe anemia and stress erythropoiesis. The integrin,β1, which partners with α4 or α5 is required for splenic erythropoiesis under the stress, but bone marrow stress erythropoiesis produces progenitor cells that are able to mobilize to the peripheral blood, although the late progenitors have maturation defects [45]. In the mouse model wherein integrin α4 is deleted, the mice experience a delayed recovery of the erythroid progenitor population in response to phenyl hydrazine and defective erythroid cells under both basal and stressed conditions [45]. When further knockout mouse models were created in which the integrins α4 or α5 were selectively removed in the erythroid lineage conditionally either early or late in erythroid development, only α4 was required for proper erythroid maturation [44]. Collectively, this work demonstrates the importance of adhesion to the ECM via integrins.

## 3. Integrins as ECM Sensors in the Context of Megakaryocytosis

MKs also conduct forces across the membrane through integrin attachment to ECM proteins. Since collagen has such a large role in ECM biomechanical properties, it has been the focus of research for the understanding of MK development and platelet formation [30,46,47]. MKs express two main receptors for collagen, integrin-α2β1 and glycoprotein VI [47]; however, MK expression of leukocyte-associated immunoglobulin-like 1 (LAIR1) and discoidin domain receptor (DDR1) that also bind collagen have been described in the literature [48,49]. Early MK progenitors (CFU-MK) have a lower affinity for adhering to collagen compared to later MK progenitors [50], which is interesting as integrin-α2β1 and glycoprotein VI expression occurs early in MK development [50,51]. It has been established that collagen I inhibits proplatelet formation (PPF) in order to prevent the release of immature platelets in the bone marrow, while collagen IV and laminin support PPF in the sinusoids [52]. To date, collagen I, III, and IV localization to a specific compartment of the bone marrow is elusive; MKs localize equally with collagen I and IV but more so with collagen IV within the vascular niche [51]. Studies utilizing antibodies and knockout mice implicate the inhibitory effects of collagen I on MK PPF by the glycoprotein VI, although there is a modest compensatory effect through integrin α2β1 [51]. In studies where ex vivo human HSPC were co-cultured with human osteoblasts (hOST), MK maturation and PPF was inhibited [46]. This effect was mediated by the fibrillar structure of collagen I and engagement through integrin α2β1 [46], and consequently activation of the Rho/Rock pathway [53]. MKs cultured on N-acetylated collagen I, which decreased mechanical tension, produced more platelets compared to unaltered collagen I [54]. This further supports the fact that the rigidity of the ECM in combination with the structural proteins affects platelet biogenesis [30,31]. Unlike collagen I, collagen III and IV promote platelet formation via the PI3K/Akt pathway [13,30]. Furthermore, primary MKs are able to self-regulate their immediate microenvironment by secreting ECM components collagen IV; fibronectin; laminin [13]; and lysyl oxidase (LOX), a copper-dependent enzyme, which controls ECM stiffness by regulating the cross-linking of collagen fibers [55]. Highly expressed in immature MKs and down-regulated in MKs of higher ploidy [56], LOX inhibition lessens BM fibrosis in the GATA-1^low^ and JAK2V671F mouse models [56,57].

The ECM component fibronectin plays a pivotal role in MK biology as it is necessary for platelet production [58], and when cultured CD34^+^ cells stimulated with thrombopoietin (TPO) were grown on a fibronectin matrix, the number of CFU-MK and percentage of CD41^+^ cells were higher compared to liquid only cultures [59]. HSPC cultures on fibronectin resulted in an increase in mouse HSC with an increase in MK output [13]. The major fibronectin binding integrins on MKs are VLA-4 (α4β1) and VLA-5 (α5β1), which are expressed throughout MK development [50,60]. HSC cultures produced more CFU-MKs on fibronectin as stiffness increased [8]. Integrin function on MKs is necessary, as a pan-blockade of integrins blocked TPO-stimulated growth; specifically neutralizing VLA-4 with monoclonal antibody blocked MK growth in TPO-stimulated bone marrow cultures [61]. In the MK cell line CHRF-288, full MK differentiation, CD41 expression, adhesion, size, polyploidy, and PPF were only achieved on cultures with fibronectin [58]. Integrins transmit mechanical signals via engagement with the ECM; these are relayed into the cells by activation of focal adhesion kinase (FAK) which subsequently activates the RhoA/ROCK pathway [62]. Activation of the RhoA/Rock mobilizes the cytoskeleton in MKs by phosphorylation of non-muscle myosin II (NMM-II) which is inhibitory to PPF [63]. Taken together, studies show that outcomes of specific integrin binding/activation depend on the matrix as well.

## 4. Mechanosensitive Ion Channels in the Erythroid Lineage

Mechanosensitive ion channels are pore forming channels that specialize in sensing physical sensations, such as force, osmotic pressure, stretching, or shear stresss, and converting the stimuli into biochemical signals through the transfer of ions across the cellular membrane [64]. There are multiple mechanosensitive channel types, such as transient receptor potential cation channels, mechanosensitive potassium channels, and Piezo ion channels [64]. These channel families differ in their structure, function, activation, and cation selectivity [64]. This review focuses on the mechanosensitive ion channels in the TRP and Piezo families, as relatively few studies to date address mechanosensitive ion channels in the context of erythroid and MK biology. In the family of mechanically activated channels known as the transient receptor potential cation channel subfamily, member 3 and 6 (TRPC3 and TRPC6) are Ca^2+^ permeable non-selective ion channels that are downstream of PLC and DAG [65]. Both TRPC3 and TRPC6 are agonist activated [65,66]. Transient receptor potential vanilloid 4 (TRPV4) also is a Ca^2+^ permeable non-selection ion channel that functions in the capacity of an osmosensor, thermosensor, and like TRPC3 and TRPC6 can be activated by agonist stimulation [67,68,69,70,71,72]. The Piezo channel family has no sequence homology to any other mechanosensitive channel family [73]. The Piezo family of proteins was identified by neuroscientist Ardem Patapoutian who named two related ion channel proteins Piezo1 and Piezo2 – from the Greek word piezein, meaning squeeze/press. Piezo family channels activate in response to indentation and stress [64], and then the influx of Ca^2+^ into the cell triggers a series of signaling events that propagate the mechanical signal.

Two members of the family of mechanically activated channels, transient receptor potential cation channel subfamily, TRPC3 and TRPC6, are present on the surfaces of human erythroid progenitors and mature RBCs, allowing the passage of calcium (Ca^2+^) through the membrane [74,75,76]. Erythroid cells and specifically RBCs are very sensitive to small changes in intracellular ion concentrations. Stimulation of erythroid TRPC3 increased intracellular Ca^2+^ in an EPO dependent manner [76,77] and is negatively modulated by TRPC6 [77]. TRPC3 mobilizes to the plasma membrane after EPO stimulation [74] possibly through TRPC3 interaction with proteins involved in the cytoskeleton [78,79]. The function of TRPC6 is unaffected by EPO stimulation, and in the mature RBC activation may be dependent on osmotic changes [75]. TRPC6 may mediate eryptosis a mechanism to clear damaged RBCs [75]. Recent reports have contested whether the family of TRP channels can directly conduct mechanical sensation, and have suggested that activation of these channels is downstream of other mechanosensitive sensors [80].

Autosomal dominant hemolytic anemia hereditary xerocytosis is linked to gain of function mutations in the mechanosensitive ion channel Piezo1 [81,82]. Piezo1 is a broadly expressed mechanosensitive ion channel responsible for transforming mechanical stimuli such as touch, pressure, shear stress, and membrane tension into a biochemical signal through the transmembrane flow of Ca^2+^ across a number of cell types that include vascular epithelial and neuronal tissue. [73,83]. In RBCs, Piezo1 transmit stimuli related to membrane stretch, [84] distortion, [85], and curvature [86]. The Piezo1 channel is non-selectively pharmacologically blocked by the spider venom inhibitor, GsMTx4 [87]. GsMTx4 intercalates into membrane lipids and alters membrane fluidity, thereby altering membrane tension and altering Piezo1 activation kinetics [87]. Activation of Piezo1 either by stretch or stimulation by Yoda-1, a Piezo1 specific agonist [88], results in robust Ca^2+^ entry [89,90] and eventual dehydration of RBCs which is similar to the phenotype seen in RBCs with gain of function Piezo1 mutations [90]. Mechanistically, the increased intracellular Ca^2+^ opens the Gardos channel (KCa3.1), which controls RBC volume [91]. Gardos channel mutations result in hereditary xerocytosis as well [92,93]. Piezo1 expression is up-regulated during the erythroid differentiation of cultured CD34^+^ HSPCs [82]. Yoda-1 activation of Piezo1 in ex vivo cultures alters the dynamics of normal erythroid differentiation by decreasing glycophorin A by 75%, altering the GATA2/GATA1 ratio, and reducing hemoglobinization [94]. Similar dynamics were observed in erythroid cells cultured from CD34^+^ HSPCs obtained from individuals with gain of function Piezo1 mutations [94]. These studies shed light on the functional characterization of the Piezo1 channel, where on the erythroid progenitor it functions to influence differentiation, but Piezo1 functions on the mature RBC to control cell volume.

## 5. Mechanosensitive Ion Channels in the Megakaryocyte Lineage

As discussed above, both the erythroid and megakaryocytic lineages detect and react to mechanical forces through integrins as well as mechanosensitive ion channels. Extracellular Ca^2+^ plays an important role in MK ability to sense the ECM, as MKs and/or MK cell lines (Meg-01) have been observed to express the mechanosensitive ion channels [30,95,96]. The mechanosensitive receptor TRPC6 has been linked to Ca^2+^ influx and platelet function [97,98,99]. Treatment of proliferating MK with 5 μM SKF96365, a TRPC6 specific inhibitor, reduced MK numbers by 50% [95]. Although linked to MK proliferation in vitro, knockout of TRPC6 in mice had no effect on platelet function either in vitro or in vivo [100]. The platelet response to activating agonists and aggregation studies conducted in vitro with platelets obtained from TRPC6 −/− mice were not significantly different than those of wild-type mice [100]. Thrombus formation after mechanical injury of the abdominal aorta was also unaffected, as were tail-bleed times for wild-type and knockout mice [100]. In MKs, the mechanosensitive ion channel, TRPV4, senses soft ECM which increases the influx of intracellular Ca^2+^, which leads to the activation of β1-integrin and Akt phosphorylation and increases platelet production [30]. Platelet production decreased with inhibition of TRPV4 on MKs cultured on collagen IV, and moreover, mice treated with LOX inhibitor (BAPN) had softer ECMs, higher platelet counts, and an increase in TRVP4 activity [30].

Piezo1 expression was found on platelets and the MK cell line, Meg-01 [96]. Piezo1 was activated in platelets and Meg-01 by fluid shear stress, and inhibition of Piezo1 with GsMTx-4 abrogated the Ca^2+^ influx [96]. Yoda-1 stimulation of platelets increased Ca^2+^ influx by over 170% [96]. In vitro thrombus formation of whole blood was reduced by GsMTx-4; however, platelet aggregation was unaffected [96]. As noted above, the role of Piezo1 function has been well-characterized in RBC physiology; Piezo1 activation by Yoda-1 alters erythroid differentiation kinetics [86,90,94]. Since erythrocytes and MKs share a common progenitor, Piezo1 may contribute to MK maturation, a role that needs to be clarified. Taken together these studies demonstrate that mechanosensitive ion channels present on the surface of MKs sense matrix rigidity and that the biomechanical tone of the ECM contributes the dynamics of MK maturation and platelet production.

## 6. Discussion

Both MKs and erythrocytes emerge from a common progenitor. Differential expression of integrins and ion channel mechanosensors in the fully differentiated cells of these lineages, as well as the ECM composition, impact their properties and courses of development, as further summarized in Table 1. In erythroid progenitors, adhesion to extracellular fibronectin by the integrins VLA-4 and VLA-5 plays a role in the dynamics of RBC production. Engagement of integrin-α2β1 and/or glycoprotein VI with collagen I compared to collagen IV influences proplatelet formation by MKs.

MK progenitors require fibronectin for platelet formation which is mediated through integrins VLA-4 and VLA-5. The mechanosensitive ion channels of the family of mechanically activated channels known as the transient receptor potential channels of the canonical type, TRPC3 and TRPC6, are present on the surfaces of erythroid progenitors and mature RBCs and mediate EPO stimulated transmembrane Ca^2+^ influx. Erythroid expression of the mechanosensitive ion channel, Piezo1, plays a role erythroid progenitor proliferation and regulates RBC volume in response mechanical pressure. The functions of mechanosensitive ion channels, TRPC6, TRPV4, and Piezo1, on MKs and platelet biology, are starting to be elucidated. Taken together, the effects of bone marrow components and the biophysical state of the bone contribute to erythroid and MK lineage development, and a greater understanding of these interactions could shed more light on mechanisms of underlying diseases.

## Figures and Tables

**Table 1:** Erythroid and megakaryocyte mechanosensors.

Mechanosensor	Comments	Reference
VLA-4 (α4β1)	Expression and attachment to fibronectin decreases as erythroid progenitors differentiate Delay in recovery from stress erythropoiesis and defective erythroid cells found in α4 knockout mice Enhances TPO stimulated MK growth	[39] [45] [61]
VLA-5 (α5β1)	Redundant role in erythroid development	[43,44]
β1-integrin	Required for stress induced splenic erythropoiesis	[45]
α2β1	Engagement with collagen I inhibits Mk maturation and PPF Binding to collagen IV promotes MK maturation and PPF	[46] [30]
Glycoprotein VI	Signals the inhibitory effect of collagen I on PPF in MKs	[51]
TRPC3	EPO dependent increase in Ca^2+^ influx in erythroid cells	[74,76,77]
TRPC6	Negatively modulates TRPC3 activity in erythroid cell Potentially mediates eryptosis Ca2+ entry into platelet; platelet activation Inhibition decreased the number of MKs in vitro	[77] [75] [97,98,99] [95]
TRPV4	Increases MK maturation and platelet formation on soft matrix, activate β1-integrin and PI3K/Akt pathway in MKs	[30]
Piezo1	Gain of function mutations cause Hereditary Xerocytosis Modulates RBC volume Role in erythroid differentiation; activation delays maturation Activated on platelets in response to shear stress; inhibition reduced in vitro thrombus formation	[81,82] [89,90] [94] [96]
